# The collection of birds from Mozambique at the Instituto de Investigação Científica Tropical of the University of Lisbon (Portugal)

**DOI:** 10.3897/zookeys.708.13351

**Published:** 2017-10-16

**Authors:** Miguel Monteiro, Rui Figueira, Martim Melo, Michael Stuart Lyne Mills3,4, Pedro Beja, Cristiane Bastos-Silveira, Manuela Ramos, Diana Rodrigues, Isabel Queirós Neves5,7, Susana Consciência, Luís Reino

**Affiliations:** 1 CIBIO/InBIO-Centro de Investigação em Biodiversidade e Recursos Genéticos, Universidade do Porto, Vairão, Portugal; 2 CEABN/InBio, Centro de Ecologia Aplicada “Professor Baeta Neves”, Instituto Superior de Agronomia, Universidade de Lisboa, Tapada da Ajuda, 1349-017 Lisboa, Portugal; 3 FitzPatrick Institute of African Ornithology, University of Cape Town, Rondebosch 7701X, South Africa; 4 P. Leventis Ornithological Research Institute, University of Jos, PO Box 13404, Jos, Plateau State, Nigeria; 5 Museu Nacional de História Natural e da Ciência, Universidade de Lisboa, Rua da Escola Politécnica 56, 1250-102 Lisboa, Portugal; 6 MARE-FCUL, DOP/UAç - Departamento Oceanografia e Pescas, Univ. Açores, Rua Prof. Dr. Frederico Machado, 9901-862 Horta, Portugal; 7 CESAM-Centre for Environmental and Marine Studies, Universidade de Aveiro, 3810-193 Aveiro, Portugal; 8 Estrada de Mem Martins n 251 1ºDto, 2725-391 Mem Martins, Sintra, Portugal; 9 CIBIO/InBIO-Centro de Investigação em Biodiversidade e Recursos Genéticos, Universidade de Évora, 7004-516 Évora, Portugal

**Keywords:** Animalia, Aves, Biodiversity databases, Chordata, museum, species occurrence data, specimen, southern Africa

## Abstract

The Instituto de Investigação Científica Tropical of the University of Lisbon, which resulted from the recent merger (in 2015) of the former state laboratory Instituto de Investigação Científica Tropical in the University of Lisbon, holds an important collection of bird skins from the Portuguese-speaking African Countries (Angola, Mozambique, São Tomé and Príncipe, Guinea Bissau and Cape Verde), gathered as a result of several scientific expeditions made during the colonial period. In this paper, the subset from Mozambique is described, which was taxonomically revised and georeferenced. It contains 1585 specimens belonging to 412 taxa, collected between 1932 and 1971, but mainly in 1948 (43% of specimens) and 1955 (30% of specimens). The collection covers all eleven provinces of the country, although areas south of the Zambezi River are better represented than those north of the river. The provinces with the highest number of specimens were Maputo, Sofala, and Gaza. Although it is a relatively small collection with a patchy coverage, it adds significantly to Global Biodiversity Information Facility, with 15% of all records available before and during the collecting period (1830–1971) being the second largest dataset for that period for Mozambique.

## Introduction

Mozambique, located along the southeast coast of Africa, has a total land area of 801,590 km^2^ encompassing three major biomes: the Afrotropical Highlands biome in the montane areas, the East African Coast biome in the lowlands, and the Zambezian biome represented by *Brachystegia* woodlands (miombo) at mid elevations (Fishpool and Evans 2001). This diversity of habitats supports a rich avifauna (Parker 1999; Ministry for the Coordination of Environmental Affairs 2009), with 671 species recorded, more than 530 of which are breeding residents (BirdLife International 2015). The country holds populations of 29 globally threatened bird species: 4 Critically Endangered, 11 Endangered, and 14 Vulnerable (IUCN 2017).

Despite the fairly high number of bird species recorded, the avifauna of Mozambique is still one of the least studied on the African continent (Borghesio et al. 2009), with several parts of the territory poorly explored, especially north of the Zambezi River (Dowsett-Lemaire 2008; Ryan and Spottiswoode 2003). The protracted Mozambican Civil War (1977–1992) was the main factor resulting in this lack of knowledge, as during that time very few expeditions or scientific studies were carried out (Parker 1999). As a result, a substantial part of current knowledge is still based on studies conducted prior to the war (Clancey 1996), represented mostly by bird skin collections kept at scientific institutions and natural history museums around the world (Frade 1950; Pinto 1953a, 1953b; Frade and Pinto 1954). After the war, there has been a renewed interest, albeit still modest, in the avifauna of Mozambique that has improved the knowledge of species diversity and distributions (e.g., Clancey 1996; Parker 1999; Spottiswoode et al 2008; Fishpool and Bayliss 2010). These recent studies have played a fundamental role in guiding national conservation strategies for the avifauna of Mozambique, which have targeted areas dominated by high-priority habitats like the sensitive forests that are home to many threatened species, the coastal zones where many migratory birds spend their non-breeding season, and freshwater habitats that are inhabited by many waterbirds (Borghesio et al. 2009; Moseley et al. 2012).

The present paper describes the dataset of the Mozambican bird skins held in the collection at the Instituto de Investigação Científica Tropical of the University of Lisbon (IICT-ULisboa) in Lisbon. This institution is a specialised unit of the University of Lisbon that resulted from the merging of almost all sections of the Instituto de Investigação Científica Tropical, a former state laboratory, in the University of Lisbon, in July 2015. This new unit shares the director with the National Museum of Natural History and Science of the University of Lisbon, and all the zoological and herbarium collections of IICT are in the process of being moved to the Museum. After the conclusion of relocation process, collections will remain open for the scientific community. The dataset is based on a full taxonomic revision of the bird specimens and georeferenced collection locations, accessed while in the facilities of the former IICT. This is the third in a series of studies based on the IICT-ULisboa collection, with the datasets of Angola (Monteiro et al. 2014) and São Tomé and Príncipe (Monteiro et al. 2016) already summarised. As with the previous datasets, the Mozambican dataset is freely available online on the IICT IPT provider (http://maerua.iict.pt/ipt) and on the Global Biodiversity Information Facility (GBIF) data portal (http://www.gbif.org).

## Importance of biological and natural history collections

For more than two centuries, specimens from different taxa have been kept as part of natural history collections in both Natural History museums and herbariums worldwide. One of the goals was to show the outstanding biological diversity, which included very valuable and rare species, to the general public while safeguarding an interest in scientific research (e.g., Pyke and Paul 2010). These biological museums have become large repositories of compiled knowledge and reference material that can be used in several different lines of research in the field of biological sciences (Winker 2004). Currently, the information gathered on the natural collections is considered critical to deal with some biodiversity conservation matters, which includes different topics from ecology, taxonomy, biodiversity loss, biological invasions, agriculture, and public health (Graham et al. 2004).

A foremost contribution of biological collections is linked to the potential of their records being used as historical databases, since the taxonomical and distribution information it contains can cover long periods and relatively large spatial scales (Hromada et al. 2015). Moreover, when the specimens’ records are combined with environmental and historical data, spatial patterns can be depicted and historical distributions can be compared with both present and future projections (e.g., Graham et al. 2004; Lavoie 2013). Each of the above data features is of extreme importance, especially in underdeveloped countries where the research means are scarce and scientific studies are limited. The use of collection records in these regions may be a cost-effective valuable resource, reducing or even eliminating the need for fieldwork in remote areas (Ponder et al. 2001).

In terms of the relevance of the IICT-ULisboa’s bird collection, albeit limited, this is a relevant contribution to Mozambique´s ornithology, a poorly studied territory mainly due to the detrimental effects of both the Mozambican war of independence (1964–1974) and its civil war (1977–1992). This collection, though relatively small, represents the second largest set of bird records for the country available through GBIF before and during the collecting period (1830–1971) which represents 15% of all the data for that same time. Some of the records are the first species record for Mozambique and represent the only preserved museum specimens in the entire GBIF´s dataset (1830–2017) (GBIF.org, 2017). This is the case of the Secretary bird (*Sagittarius serpentarius* (Miller JF, 1779)) and the White-headed Vulture (*Trigonoceps occipitalis* (Burchell, 1824)). Furthermore, some data from the least-explored provinces of Niassa, Cabo Delgado, and Nampula, and from many biome-restricted species, are also added to the database.

Despite the critical value of this kind of data, the information available in many collections is still restricted as they are not easily accessible or were not even seen by specialists (Ponder et al. 2001). Therefore, it is highly recommended to facilitate the accessibility to data from as many collections as possible, which would increase the number of sources that can be reached by the scientific community (Suarez and Tsutsui 2004). One of the best ways to increase that availability is through the Internet by the digitization of the records on open access online platforms (Winker 2004). Another relevant issue that requires our attention is the declining support for taxonomic and systematic research by constituencies (e.g., Gropp 2003). This decline has already resulted in large budget cuts in many museums throughout the world, and is putting the use of biological collections at risk (Dalton 2003). As a result, it is vital to contribute to the preservation of biological collections and increase the awareness for a major visibility and access to them.

## General description

The IICT-ULisboa holds a collection of 5598 African bird specimens/skins (hereafter “the collection”), mainly from Angola, Cape Verde, Guinea Bissau, Mozambique and São Tomé and Príncipe. The dataset in this paper is a subset of the collection, and contains all specimens from Mozambique, the largest subset for any country represented in the collection (hereafter “the dataset”). The dataset comprises 1585 specimens from 412 taxa, 25 orders and 79 families, which were fully taxonomically revised and georeferenced. Specimens were collected between 1932 and 1971 from 197 different locations, although 73% of all Mozambican specimens were collected in 1948 (43%) and 1955 (30%). Most of these specimens were collected during expeditions of the Missão Zoológica de Moçambique, from south of the Zambezi River, largely because these are the most accessible regions of the country. Of the many collectors, the most significant contributions were made by António da Rosa Pinto and Rui Quadros (118 specimens each) and Mussolini Fajardo (46 specimens). All of them were members of the Museu Álvaro de Castro (now Museu de História Natural, Maputo, Mozambique) and participants on the expeditions of the Missão Zoológica de Moçambique.

## Records of a special significance

The collection contains specimens of five of the 29 globally threatened species found in Mozambique (Fishpool and Evans 2001). They all belong to the order Accipitriformes and include two vulnerable species – Martial Eagle (*Polemaetus bellicosus* (Daudin, 1800)), Secretary bird (*Sagittarius serpentarius* (Miller, JF, 1779)) – and three critically endangered species – Hooded Vulture (*Necrosyrtes monachus* (Temminck, 1823)), White-backed Vulture (*Gyps africanus* Salvadori, 1865) and White-headed Vulture (*Trigonoceps occipitalis* (Burchell, 1824)).

The collection holds representatives of five of the 30 biome-restricted species of the Afrotropical Highlands biome found in Mozambique, seven of the 24 species from the East African Coast biome, and 12 of the 26 species of the Zambezian biome (Parker 1999) (Table 1). Several species for which there are few records for the country are represented in the collection, such as Black-rumped Buttonquail (*Turnix nanus* (Sundevall, 1850)), African Blue Flycatcher (*Elminia longicauda* (Swainson, 1838)), Groundscraper Thrush (*Turdus litsitsirupa* (Smith A, 1836)) and Miombo Scrub Robin (*Cercotrichas barbata* (Hartlaub and Finsch, 1870) (e.g., GBIF.org 2017). The collection also includes some of Mozambique´s biome-restricted species of particular interest such as Anchieta’s Sunbird (*Anthreptes anchietae* (Barboza du Bocage, 1878)) and Miombo Double-collared Sunbird (*Cinnyris manoensis* Reichenow, 1907) from the Zambezian biome. Both species have considerably restricted distributions in Southern Africa, being relatively uncommon in parts of their range (BirdLife 2016a, b) and the near threatened Neergaard’s Sunbird (*Cinnyris neergaardi* Grant CHB, 1908) from the East African Coast biome. This species is mainly restricted to Mozambique and restricted area in South Africa. Its conservation status reflects concerns about the moderately small population, which may be declining owing the clearance of its native forest habitats (BirdLife 2016c).

**Table 1.** Biome-restricted species (Parker 1999) that occur in Mozambique and are represented in the IICT-ULisboa collection. The taxonomic nomenclature follows the International Ornithological Council Bird List v6.1 (Gill and Donsker 2016).

Common Name	Scientific Name	N	IUCN Red List (version 2017)	Biome
Racket-tailed Roller	*Coracias spatulatus* Trimen, 1880	3	Least concern	Zambezian
Mangrove Kingfisher	*Halcyon senegaloides* Smith A, 1834	1	Least concern	East African Coast
Dickinson's Kestrel	*Falco dickinsoni* Sclater PL, 1864	2	Least concern	Zambezian
Brown-headed Parrot	*Poicephalus cryptoxanthus* (Peters W, 1854)	5	Least concern	East African Coast
Pale Batis	*Batis soror* Reichenow, 1903	5	Least concern	East African Coast
Olive Bushshrike	*Chlorophoneus olivaceus* (Shaw, 1809)	2	Least concern	Afrotropical Highlands
White-tailed Crested Flycatcher	*Elminia albonotata* (Sharpe, 1891)	2	Least concern	Afrotropical Highlands
Stripe-cheeked Greenbul	*Arizelocichla milanjensis* (Shelley, 1894)	4	Least concern	Afrotropical Highlands
Black-bellied Starling	*Notopholia corrusca* (Nordmann, 1835)	8	Least concern	East African Coast
Meves's Starling	*Lamprotornis mevesii* (Wahlberg, 1856)	3	Least concern	Zambezian
Miombo Scrub Robin	*Cercotrichas barbata* (Hartlaub & Finsch, 1870)	2	Least concern	Zambezian
Kurrichane Thrush	*Turdus libonyana* (Smith A, 1836)	16	Least concern	Zambezian
White-throated Robin-Chat	*Cossypha humeralis* (Smith A, 1836)	6	Least concern	Zambezian
White-starred Robin	*Pogonocichla stellata* (Vieillot, 1818)	3	Least concern	Afrotropical Highlands
Miombo Rock Thrush	*Monticola angolensis* Sousa, 1888	16	Least concern	Zambezian
Arnott's Chat	*Myrmecocichla arnotti* (Tristram, 1869)	1	Least concern	Zambezian
Anchieta's Sunbird	*Anthreptes anchietae* (Barboza du Bocage, 1878)	1	Least concern	Zambezian
Grey Sunbird	*Cyanomitra veroxii* (Smith A, 1832)	2	Least concern	East African Coast
Miombo Double-collared Sunbird	*Cinnyris manoensis* Reichenow, 1907	3	Least concern	Zambezian
Neergaard's Sunbird	*Cinnyris neergaardi* Grant CHB, 1908	1	Near threatened	East African Coast
White-bellied Sunbird	*Cinnyris talatala* Smith A, 1836	12	Least concern	Zambezian
Red-faced Crimsonwing	*Cryptospiza reichenovii* (Hartlaub, 1874)	1	Least concern	Afrotropical Highlands
Pink-throated Twinspot	*Hypargos margaritatus* (Strickland, 1844)	3	Least concern	East African Coast
Black-eared Seedeater	*Crithagra mennelli* (Chubb EC, 1908)	1	Least concern	Zambezian

## Taxonomic coverage

**Figure 1.**
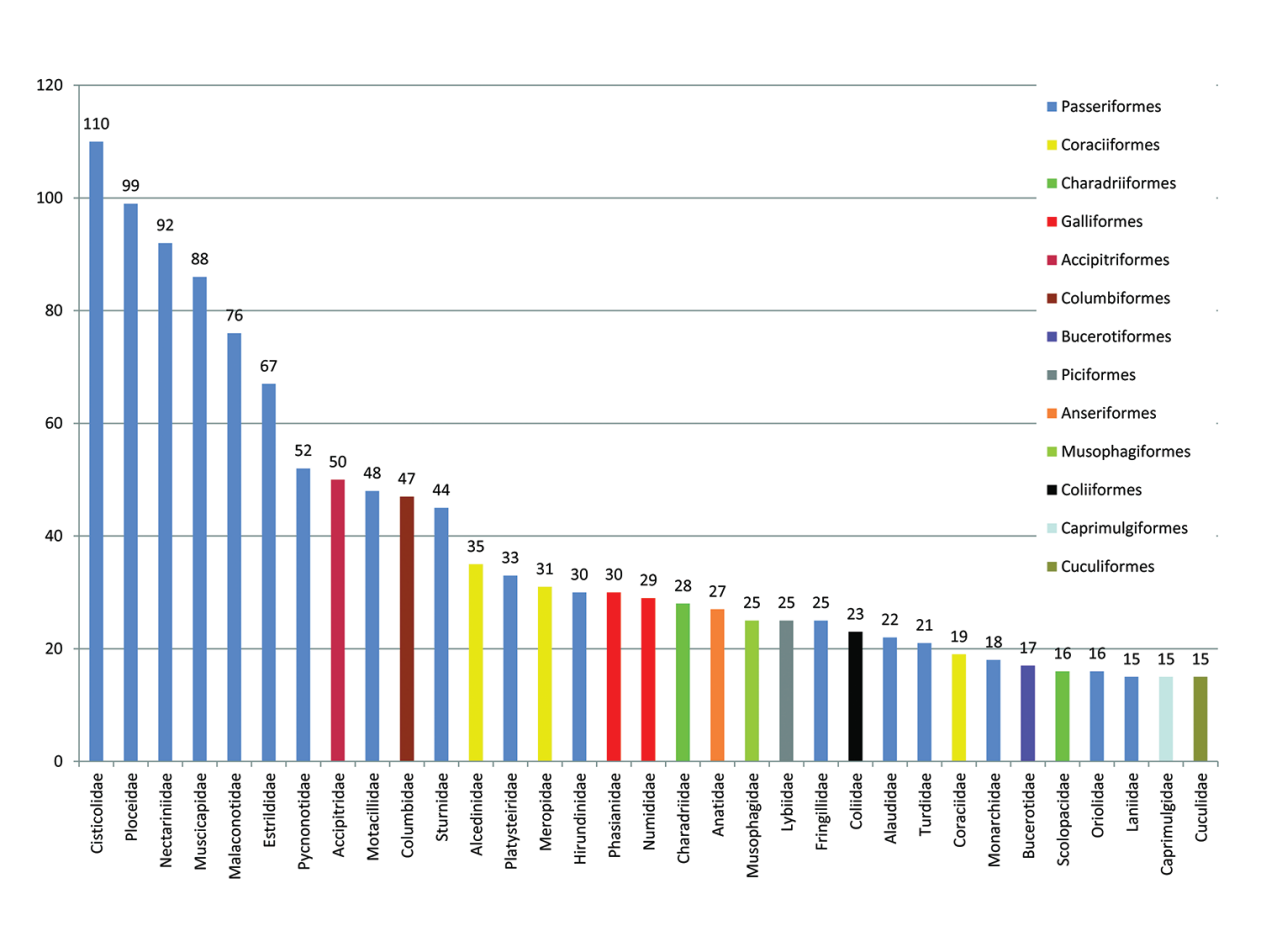
Total number of bird specimens from Mozambique, per family, held in the zoological collections of IICT-ULisboa (Lisbon). The legend lists the corresponding Orders, with assigned colours. Only the categories of families having 15 or more specimens are labelled.

The dataset includes specimens from 25 orders and 79 families. Passeriformes are by far the best-represented order (63% of the specimens), followed by Coraciiformes (5.4%) and Charadriiformes (4.9%) (Figure 1). The Cisticolidae, Ploceidae and Nectariniidae are the families with most records (110, 99 and 92 specimens, respectively).

### Taxonomic ranks

**Kingdom: Animalia**

**Phylum: Chordata**

**Class: Aves**

**Order**: Accipitriformes, Anseriformes, Apodiformes, Bucerotiformes, Caprimulgiformes, Charadriiformes, Ciconiiformes, Coliiformes, Columbiformes, Coraciiformes, Cuculiformes, Falconiformes, Galliformes, Gruiformes, Musophagiformes, Otidiformes, Passeriformes, Pelecaniformes, Piciformes, Podicipediformes, Psittaciformes, Pterocliformes, Strigiformes, Suliformes, Trogoniformes

**Family**: Accipitridae, Acrocephalidae, Alaudidae, Alcedinidae, Anatidae, Anhingidae, Apodidae, Ardeidae, Bucerotidae, Burhinidae, Campephagidae, Caprimulgidae, Certhiidae, Charadriidae, Ciconiidae, Cisticolidae, Coliidae, Columbidae, Coraciidae, Corvidae, Cuculidae, Dicruridae, Emberizidae, Estrildidae, Eurylaimidae, Falconidae, Fringillidae, Glareolidae, Hirundinidae, Hyliotidae, Indicatoridae, Jacanidae, Laniidae, Laridae, Locustellidae, Lybiidae, Macrosphenidae, Malaconotidae, Meropidae, Monarchidae, Motacillidae, Muscicapidae, Musophagidae, Nectariniidae, Nicatoridae, Numididae, Oriolidae, Otididae, Paridae, Passeridae, Pelecanidae, Phalacrocoracidae, Phasianidae, Phoeniculidae, Platysteiridae, Ploceidae, Podicipedidae, Psittacidae, Pteroclidae, Pycnonotidae, Rallidae, Remizidae, Sagittariidae, Scolopacidae, Scopidae, Stenostiridae, Strigidae, Sturnidae, Sylviidae, Threskiornithidae, Timaliidae, Trogonidae, Turdidae, Turnicidae, Tytonidae, Upupidae, Vangidae, Viduidae, Zosteropidae

**Common names**: Birds

### Spatial and temporal coverage

**Figure 2.**
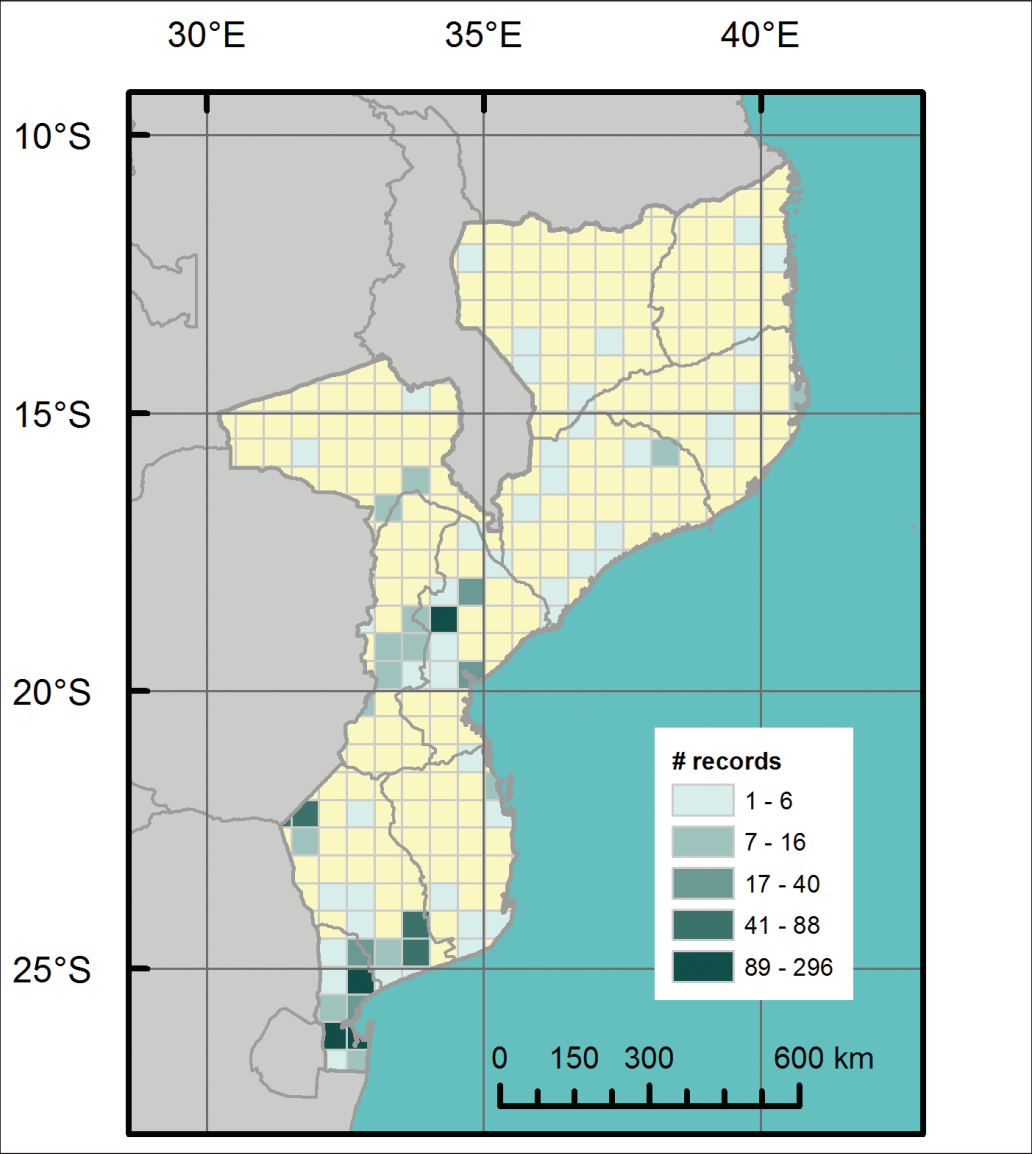
Distribution map of the locations of specimens’ occurrence throughout the territory of Mozambique held in the zoological collections of IICT-ULisboa (Lisbon).

**General spatial coverage**: The collection covers all eleven provinces of Mozambique, although the distribution of the records is uneven between them (Figure 2). From fewest to most specimens, the provinces are represented as follows: Cabo Delgado (2), Niassa (5), Nampula (5), Tete (18), Zambésia (24), Inhambane (35), Manica (39), Gaza (247), Sofala (353), and Maputo (841). The name “Maputo” refers both to Maputo and Maputo City provinces. There are 12 specimens collected from a locality that we were unable to geo-reference, and six specimens with no locality given.

**Coordinates**: Mozambique (10°S and 27°S Latitude; 30°E and 41°E Longitude)

**Figure 3.**
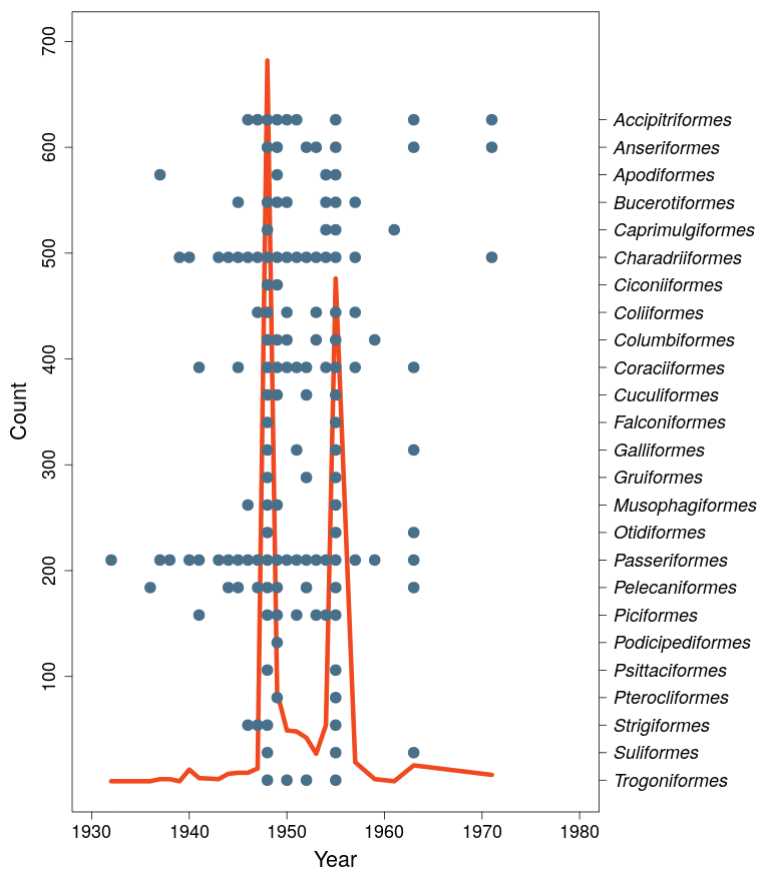
Temporal profile of the sampling leading to bird skin collection held at the zoological collections of IICT-ULisboa (Lisbon). Blue dots represent sampling years for each Order.

**Temporal coverage**: The temporal range of the records is between 1932 and 1971 (Figure 3). Two main peak periods are 1948 and 1955, which together represent 73% of the collected specimens and correspond to the dates of the Missão Zoológica de Moçambique´s expeditions.

## Methods

**Method step description**: During the ARCA project (2008–2010), the mammal and bird collections of the IICT were initially catalogued with the use of the software Specify Workbench, and later all that information was imported to Specify version 6 (Specify Software Project 2013). At that time, there were no taxonomic experts available to check the collections, and so the imported data were directly copied out from the labels without making any corrections or taxonomic updates. In 2015–2016, the collection was revised by the first author, following the procedures of the previous works on the collection (Monteiro et al. 2014, Monteiro et al. 2016).

A revision of the database was made in 2015–2016 based on the IOC Bird List v6.1 (Gill and Donsker 2014), and all the original information of each bird specimen (collector, date of collection, collection locality, and descriptions of bare parts) was double-checked to avoid transcription errors. As georeferenced location information was not available on specimen labels or associated book manuals, specimens were georeferenced according to the guidelines of Chapman and Wieczorek (2006). The geographical gazetteer Geolocate was primarily used to determine location coordinates for collection localities, with further data gleaned from sources such as Google Maps, IICT´s botanical geodata, and a series of 1:250000 maps for Mozambique. Geographical coordinates are given in decimal degrees, based on datum WGS 84. For ten records it was not possible to determine coordinates due to incomplete information.

**Study extent description**: The study covers all eleven provinces of Mozambique, although the southern (1123 records) and central provinces (410 records) of the country are much better represented than the northern provinces (36 records). The provinces of Maputo, Sofala, and Gaza are the best-represented.

**Sampling description**: All records in the database come from scientific visits carried out between 1932 and 1971. The most significant contributions were made in 1948 and 1955, during expeditions of the Missão Zoológica de Moçambique. In 1948, Fernando Frade (director of the Center of Zoology of the Junta das Missões Geográficas e Investigações Coloniais) coordinated the first and major expedition of the zoological mission with the collaboration of the Museu Doutor Álvaro de Castro and the Centro de Investigação Científica Algodoeira that were both based on Mozambique. The aim of the six-month mission (June to November) was to evaluate the state of the country´s fauna. Two scientific teams (Brigada Entomológica and Brigada do Chefe da Missão) bringing together many different specialists surveyed the Mozambican territory along 12 different itineraries. In terms of ornithological results, 718 bird specimens were collected, although only 677 specimens are currently present in the collection. All bird data was published later in 1951 in two different publications of the same institution titled “Trabalhos da Missão Zoológica de Moçambique: Aves coligidas na Missão Zoologica de Moçambique” (Frade 1951) and “Trabalhos da Missão Zoológica de Moçambique: Catálogo das aves de Moçambique” (Frade and Bacelar 1951). In 1955, António Augusto da Rosa Pinto, another member of the Zoological Mission of Mozambique and the director of the Museum Doutor Álvaro de Castro, did some minor expeditions through the south region of Mozambique and Gorongosa to study the avian diversity and collect some bird specimens. The work led to a publication on the birds of Gorongosa (Pinto 1961).

**Quality control description**: The initial digitalized information that was directly transcribed from the specimen’s labels to Specify 6 was fully revised by Miguel Monteiro. This included a taxonomic revision following the IOC Bird List version 6.1 (Gill and Donsker 2016). Additionally, all the collection localities were georeferenced using the recommended processes of Chapman and Wieczorek (2006), which included the uncertainty determination of the coordinates when no substantial information was available.
